# Challenges and solutions to cancer-related financial toxicity according to Australian health professionals: qualitative results from a national survey

**DOI:** 10.1007/s00520-023-07875-4

**Published:** 2023-07-04

**Authors:** Jordana McLoone, Raymond J. Chan, Megan Varlow, Kate Whittaker, Daniel Lindsay, Carla Thamm, Lillian Leigh, Laura Muir, Gillian Mackay, Deme J. Karikios, Lee Hunt, Kim Hobbs, David E. Goldsbury, Doreen Nabukalu, Louisa G. Gordon

**Affiliations:** 1 Behavioural Sciences Unit, Kids Cancer Centre, Sydney Children’s Hospital NSW, Sydney, New South Wales Australia; 2 Discipline of Paediatrics & Child Health, UNSW Medicine & Health, Randwick Clinical Campus, University of NSW, Sydney, New South Wales Australia; 3 Caring Futures Institute, College of Nursing and Health Sciences, Flinders University, Bedford Park, South Australia Australia; 4 Cancer Council Australia, Sydney, New South Wales Australia; 5 Faculty of Medicine, University of Queensland, Brisbane, Herston, Australia; 6 QIMR Berghofer Medical Research Institute, Population Health Department, Brisbane, Herston, Australia; 7 Rare Cancers Australia, Bowral, New South Wales Australia; 8 Cancer Council NSW, Sydney, New South Wales Australia; 9 Clinical Oncology Society of Australia, Sydney, New South Wales Australia; 10 Department of Medical Oncology, Nepean Hospital, Kingswood, New South Wales Australia; 11 Sydney Medical School, University of Sydney, Sydney, New South Wales Australia; 12 Cancer Voices NSW, Sydney NSW, Australia; 13 Oncology Social Work Australia & New Zealand/Westmead Hospital NSW, Westmead, New South Wales Australia; 14 The Daffodil Centre, University of Sydney, Cancer Council NSW, Sydney, New South Wales Australia; 15 Queensland University of Technology (QUT), School of Nursing and Cancer and Palliative Care Outcomes Centre, Brisbane, Kelvin Grove, Australia

**Keywords:** Financial toxicity, Telehealth, Financial counselling

## Abstract

**Purpose:**

To qualitatively explore Australian healthcare professionals’ perspectives on how to improve the care and management of cancer-related financial toxicity, including relevant practices, services, and unmet needs.

**Methods:**

We invited healthcare professionals (HCP) who currently provide care to people with cancer within their role to complete an online survey, which was distributed via the networks of Australian clinical oncology professional associations/organisations. The survey was developed by the Clinical Oncology Society of Australia’s Financial Toxicity Working Group and contained 12 open-ended items which we analysed using descriptive content analysis and NVivo software.

**Results:**

HCPs (*n* = 277) believed that identifying and addressing financial concerns within routine cancer care was important and most believed this to be the responsibility of all HCP involved in the patient’s care. However, financial toxicity was viewed as a “blind spot” within a medical model of healthcare, with a lack of services, resources, and training identified as barriers to care. Social workers reported assessment and advocacy were part of their role, but many reported lacking formal training and understanding of financial complexities/laws. HCPs reported positive attitudes towards transparent discussions of costs and actioning cost-reduction strategies within their control, but feelings of helplessness when they perceived no solution was available.

**Conclusion:**

Identifying financial needs and providing transparent information about cancer-related costs was viewed as a cross-disciplinary responsibility, however, a lack of training and services limited the provision of support. Increased cancer-specific financial counselling and advocacy, via dedicated roles or developing HCPs’ skills, is urgently needed within the healthcare system.

**Supplementary Information:**

The online version contains supplementary material available at 10.1007/s00520-023-07875-4.

The financial costs of cancer care are increasing rapidly and cancer-related financial toxicity (FT) has been reported globally across all models of healthcare, including publicly funded healthcare countries [1], the USA [2], and low income countries [3]. FT is the negative patient-level impact of the cost of cancer [4]. It is the combined impact of direct out-of-pocket costs and indirect costs and the changing financial circumstances of an individual and their household due to cancer, its diagnosis, treatment, survivorship, and palliation. It can cause both physical and psychological harms and affect decisions which can lead to suboptimal cancer outcomes [5].

Since the term “financial toxicity” was coined in 2013 [6], many studies have described the types of expenses and risk factors which contribute to FT, as well as measured (in absolute and relative terms) the extent of FT among cancer patients and survivors [1]. However, historical data is becoming rapidly outdated with the emerging use of new oral agents, immunotherapies, precision medicine, and telehealth, each of which have greatly altered treatment pathways, outcomes, and costs [7, 8], leaving both patients and clinicians to navigate a new, and in flux, financial landscape. For example, new technologies, tests, and treatments, especially those not yet listed by insurance companies or government subsidy programs, are greatly increasing the on-treatment costs to patients. For many cancer patients treated with precision medicine, living-with cancer is increasingly common and has led to the reconfiguring of cancer as a chronic illness, with long-term healthcare costs during the survivorship period and impacts on returning to employment [9]. For others, advances in treatment and reductions in toxicity have allowed them to remain in, or return to the workforce sooner, and the rapid uptake of telehealth since the COVID-19 pandemic has reduced many patient-borne costs of attending in-person clinic appointments. Both patients and healthcare professionals (HCPs) have been thrust into a rapidly changing environment of care and costs, with HCP training and information provision not keeping pace.

Despite traditional and emerging complexities, consideration of cancer-related FT risks, advising patients of these risks, and managing these risks remains fundamental to the practice of oncology. The mitigation of FT is critical as it not only places economic hardship on individuals and their families, but may lead to psychological distress and suboptimal coping behaviours, including poor treatment adherence, or treatment abandonment, delayed care seeking, missed appointments, prioritising employment above optimal health-related decisions, and failure to purchase prescribed medicines [10–12]. These behaviours can then lead to poorer health outcomes and early mortality [13, 14], as patients make health decisions based on their needs to remain employed. Financial distress has been reported by patients as more severe than physical, social, or emotional distress [15, 16] and patients consistently report a desire for discussions of direct and indirect cancer-related costs with their care team [17–19]. Such financial discussions have the potential to reduce patient costs [20], increase treatment compliance [21], and improve clinician-patient rapport [22]. However, a recent review by Shih et al. [19] found that on average, only 27% of patients reported having had discussions about treatment costs with their HCPs.

While there is a growing body of literature in this area, studies have predominantly reported descriptive, quantitative outcomes, such as the costs of care and the frequency of FT screening, and cost discussions [1, 3, 15], with few reporting constructs related to care and services, or the underlying drivers related to HCPs’ barriers to raising cost discussions, or what strategies they use once FT is recognised [23]. Studies typically report oncologist-patient interactions, rather than the perspectives of the multidisciplinary team, including nursing, social work, and general practitioners, despite these HCPs playing a major role in patient supportive care. To address this gap, we surveyed a range of Australian HCPs to investigate the underlying factors that sustain and impede the provision of financial discussions and care/management as part of usual cancer care.

## Methods

### Participants and recruitment

We invited Australian HCPs who regularly work directly with cancer patients (defined as at least 10% of their working time) to participate in this study. To ensure national coverage, the inclusion of a wide range of health care professions, and an adequate sample size, eight national cancer organisations were asked, and agreed, to disseminate the study information/invitation and online survey link, including the Clinical Oncology Society of Australia (COSA), Cancer Nurses Society of Australia, Oncology Social Workers of Australia and New Zealand, Trans-Tasman Radiation Oncology Group, Haematology Society of Australia and New Zealand, the Australian and New Zealand Children’s Haematology and Oncology Group, the Medical Oncology Group of Australia, and Cancer Council Australia.

### Design and analysis

The COSA Financial Toxicity Working Group developed a survey based on an extensive review of the literature and expertise of the working group, which includes representation from clinical oncology, social work, nursing, behavioural sciences, law, as well as consumers and community groups. The survey was constructed using the REDCap platform and pretested for content and face validity. Given the survey was targeting busy HCPs, it was designed to be brief (10–15 min) to complete. The quantitative responses from this survey have been reported elsewhere [24]. Here, we report a qualitative evaluation of the 12 open-ended responses questions which are listed in Appendix A. Open-ended items were collated and coded using NVivo 12 software. After immersing in the data, the first author created codes based on an a priori knowledge of concepts identified in the literature and concepts which emerged during the analytical process. Through the coding process JM systematically and rigorously categorized the transcribed text. These codes were then organised into categories and themes to allow for interpretation of the data and the coherent reporting of findings [25].

### Ethics

The study was approved by the QIMR Berghofer Human Research Ethics Committee (P3792). The landing page of the survey provided participant information and a consent option. All survey responses were non-identifiable, and a screening question (do you spend at least 10% of your time, providing direct care to cancer patients or survivors?) was inserted to ensure all participants met the eligibility criteria before proceeding to survey items. The survey was anonymous and the snowballing circulation of the survey did not allow for the calculation of a response rate.

### The Australian context

Australian healthcare is provided as a mixture of both public and private sector funding. The Medicare benefits scheme (MBS) and the pharmaceuticals benefits scheme (PBS) (collectively referred to herein as Medicare) is a national, universal, tax-funded health payment scheme. Public hospital care is provided at no cost to patients and Medicare highly subsidises prescribed pharmaceuticals and most other out-of-hospital medical services such as primary care and imaging. Primary care, specialist care, and allied health services are typically covered publicly by Medicare, though when provided in private practice, there is a patient-borne gap payment. Some newer drugs, or drugs considered not acceptably cost-effective by the Australian government, are not listed on the PBS and the patient must pay for these in full.

## Results

In total, 277 surveys were completed by HCPs across Australia (all states and territories except the Northern Territory), with 232 participants responding to the open-ended items. Of those who responded to the open items, multidisciplinary representation was high, with approximately equal representation from medical specialists (e.g., oncologists) (25%), social work (26%), nursing (30%), and other professionals (e.g., allied health) (19%). Most respondents were female (80%) and worked mostly in a government-funded setting (72%).

### Who raises financial concerns?

While some clinicians responded that they were “mindful to raise it with all my patients”, others reported that they raised financial concerns only under certain circumstances, “when patients and/or carers have stopped current employment during and post treatment.” Clinicians’ most common motivation for raising financial concerns was to understand barriers to treatment adherence (“I raise it to put it on the table so I can understand potential barriers.”), patient preferences (“When delivering a fee chat, I always confirm with the patient whether or not they’re comfortable with the costs and offer options to ensure flexibility is provided”), and ensure informed consent, (“Costs are discussed by me when consenting”). In contrast, social workers reported that this was a fundamental component of their role in cancer care, “I’m a social worker with specific focus on psychosocial impacts of cancer and finances are a standard area to explore in assessing client needs and priorities.” Nurses reported that their front-line position meant they were often the most accessible staff-member to discuss financial concerns with, “Patients often raise financial concerns with the first point of contact, who is often the RN [registered nurse] assessing them at intake.” Many HCPs reported that it was important they initiated conversations, as many patients were reluctant to do so, “Many [patients] don’t due to embarrassment, shame, lack of awareness regarding the help available.”

### Whose role is it to help patients manage financial concerns?

Some HCPs reported a preference for a more siloed approach to patient care, with clinicians focusing solely on cancer diagnosis and treatment, with referral to other HCPs in cases of financial concern, “I think the focus during a consultation is on the cancer and treatment. Consultants need a referral service for patient financial burden.” However, more commonly, participants reported that FT was a cross-disciplinary issue, and all HCPs should be responsible for providing support to concerned patients, “All caregivers who come into contact with the patient at any stage.” Social work was perceived to be the profession with the greatest expertise in providing counselling for financial distress and most able to provide the most comprehensive practical advice, “Any healthcare professional, but social workers probably have the most time and knowledge to address it”. Non-social worker HCPs often reported that after identification of the issue, referral to social work was the most appropriate solution, “I think the lead clinician does have a responsibility to identify financial toxicity, but interventions/assistance is best led by a social worker.”

Social workers responded positively regarding their responsibilities to provide support to patients experiencing FT; however, they expressed concern regarding their limitations in terms of time and expertise, “We have high caseloads and there are times the financial concerns are complex. There needs to be more agencies to refer clients to for financial counselling.” Lack of specific training in this area was also raised, “It’s always been a part of my role in social work practice, although ironically not one that I have ever received formal training around.”, specifically regarding superannuation “Social workers need more training about superannuation.” and immigration, “In some cases, financial concern is due to immigration issues—supporting a relative on a bridging visa or sponsoring a spouse. Social workers need more training on immigration issues.” A lack of social work positions in outpatient departments and rural centres was also reported as a major concern and prohibitive of providing adequate and equitable access to financial counselling and support services.

### Discomfort discussing financial concerns

Discomfort discussing FT was often related to HCPs lack of solutions to resolve patient hardship, “I often feel as though it can be a challenging topic to raise and this is purely because there is such little financial support for clients, that by raising the topic would insinuate I have the answers and solutions to their financial stressors when in fact I do not. I want to help, but lack the resources.” Lack of solutions often led to a sense of helplessness and great discomfort, “Whilst I am comfortable raising financial concerns, the difficulty arises when the help is not “out there” and these patients may not be eligible or have exhausted their entitlements. That can be hard to sit with, as people often feel desperate at these times.”

Solutions offered by HCPs to combat discomfort included the normalisation of financial discussions, “Make it routine, have phrases which normalise financial concerns so they don’t feel like a stigma”; raising the topic early, “It has always been part of my consultation and having raised it, this makes it easier to address at any time in the care pathway.”; thinking about the issue from the patient’s perspective, “People who are struggling are usually grateful for acknowledgment and support.”; and taking a leadership role in the discussion, “If I am comfortable, then it makes my patients comfortable to talk to me about concerns.”

### Other barriers to addressing financial concern

Additional barriers that were commonly reported by HCPs included patient readiness, “Readiness of patients to discuss this especially when initially overwhelmed by diagnosis & treatment decision making—timing is important”; case complexity, “If the case is overly complex financial toxicity may become lost.”; lack of clinic time; lack of, or long wait times for social work referrals; and some providers failing to provide clear information regarding their fees. More broadly, HCPs reported that one of the greatest barriers to reducing FT was simply that the issue was under-recognised within a medical model of healthcare, “Financial toxicity …is the blind spot for many medical staff who just think 'medical model' and not the social model of health.”

### Information needs

HCPs listed several unmet information needs, including information regarding the services available to provide support, “I would like more information about services available” especially when social work was not available within the service, “In the outpatient setting, access to social work is limited at my service. More information about where to direct patients to for assistance outside of hospital social workers would very valuable”; how to navigate Centrelink (national social security/government income support organisation), “Information about Centrelink and appropriate application forms”; the nuanced ways FT can affect patients, “Information on the subtle ways cancer can have a financial impact. A checklist to refer to would help us all!”; and country-specific research, “Access to nationally relevant research findings about financial toxicity to help me understand my own practice experience.”

The unmet information needs of patients were also raised by HCPs who reported that often, sufficient information was not provided to patients in a transparent or timely manner. HCPs reported that all cancer patients should be informed about ongoing out-of-pocket costs, the cost differences between private and public care, the option (if available) to change from private to public at any time, and the lack of evidence for some high-cost treatments. “My biggest issue …is the ongoing out-of-pocket costs for medical care in the private sector, which is often not disclosed until after consultations or treatments.” In addition, it was reported that greater information is needed by patients to self-navigate the complex pathways and paperwork associated with obtaining financial support, “The hoops for patients to jump through have become more numerous and difficult over time. Even those people who have paid enormous policy costs to private insurers have to prove themselves every step of the way. Often until they die. It is unjust.”

### Supportive strategies identified by healthcare professionals

The strategies identified by HCPs to reduce FT typically promoted either emotion-focused, or resource-focused coping. Some HCPs aimed to increase emotional support and reduce patient distress, “[I provide] counselling around distress experienced, education on the normality of financial toxicity in cancer care.” Other HCPs reported strategies that sought to provide additional resources or assist patients to access practical supports, including offering to bill Medicare directly (the Australian universal healthcare scheme), reduce fees, defer fees, provide flexible payment plans, or request leniency on accounts. HCPs also reported actively supporting or advocating for their patients by applying for financial and other practical support services, appealing to charities/government agencies, providing a letter to support financial requests, advocating or providing public treatment options, referring to social workers (or other), spending time with the patient to explore all the options which they may not have considered, or searching for other options (e.g., small business grants, farm subsidies, financial counsellors).

## Discussion

This qualitative study provides new insights into multidisciplinary HCPs’ views of FT within cancer care. Overall, identifying and addressing FT was seen as an important aspect of cancer care. However, concerns regarding barriers such as lack of time, available services, and cancer-specific financial training were commonly cited, reflecting international research [2]. Encouragingly, discussions of FT were seen as a cross-discipline responsibility, acknowledging the different support each professional could contribute (e.g., financial concerns impacting treatment choices and adherence were best discussed with the oncologist, while counselling for distress was seen as within the social worker’s remit). Concerningly, many HCPs felt that they had limited information and options when it came to providing ideal levels of support, highlighting education and service development as critical next steps.

Acknowledging the difficulty of, and addressing HCPs reluctance to open a discussion with patients for whom they feel they have “no solution” is critical. Recent research has shown that oncologists are at risk of burnout and compassion fatigue [26], and as such, it may be detrimental to oncologists’ own mental health to expect them to provide support in an area of cancer care that is not within their primary training, or which is not adequately resourced. It is critical for all HCPs to receive training in how to navigate difficult financial discussions, as well as how to manage the emotional consequences of being confronted by distressed patients for whom they cannot provide further assistance. While a patient app is in development (DISCO App [27]), the authors are unaware of any interventions designed purposely for HCPs to effectively manage the cancer-related financial concerns of their patients, or their own coping skills. From the perspective of improving patient care and safeguarding professionals’ wellbeing, this is a critical step needed to advance the field.

Advances in intervention design and clinical care may be guided by study findings, which include the introduction of FT as a standard topic of discussion early within the cancer care trajectory, using language that normalises FT. Interventions designed to reduce HCPs reluctance to hold FT discussion could focus on increasing their belief that patients are appreciative of the topic being raised, even if no solution is reached and that validation, normalisation, and reduction of distress are positive outcomes irrespective of whether a financial solution has been found.

Training is also needed for social workers (or other relevant staff including nurses and care coordinators) to better understand and help patients to navigate government social security organisations, superannuation, and immigration rules/laws. Many social workers reported that they had not received any formal training in financial assessment and that their knowledge was limited to what they had picked up on the job. Importantly, this education and training should include when it is necessary to refer a patient to an external support, how to make an appropriate referral, and to whom. Training HCPs to make the right referrals at the right time also acknowledges that while they may be seen as best placed in a clinical setting to support patients, they have reported lack of time and knowledge as key barriers. Furthermore, both patients and clinicians need to understand the boundaries of these roles, within which it is not appropriate to provide financial or legal advice. Appropriate referrals to other professionals including free legal and financial support services, in situ health justice partnerships, financial counsellors, financial advisers, lawyers, and other professionals are essential. Given the potentially long-term impact of poor or delayed financial decisions and missed financial opportunities, adequate training and prompt referral are integral to ensure patients are provided with sound guidance, information, and support regarding financial options. Interventions and/or scalable training modules, appropriate for national roll-out and tailorable to the local context, are crucial to upskill the workforce in a rapidly changing medical and economic context.

Financial counselling services provided by state cancer services (e.g. Cancer Council NSW and Cancer Council Victoria) serve as examples of support that could be replicated. These specialised financial counsellors provide information, support, and guidance to address immediate financial concerns, but importantly, they also empower patients and their families by building financial capability and resilience, helping them to better manage the cost of cancer over the longer term.

In terms of patient empowerment, HCPs need to provide transparent and timely information regarding both direct and out-of-pocket costs related to all tests, treatments, consults, and foreseeable life-impacts (e.g., employment) [28]. This should not be limited to on-treatment costs but should continue to be made available after treatment and within palliative care/bereavement. Carers are often equally impacted (especially parents of childhood cancer patients/survivors [29]), and the financial impact of providing care for a loved one should always be acknowledged. Finally, all patients should be educated and empowered, irrespective of their known or assumed wealth, and all costs should be acknowledged as burdensome (even very small costs), as there is a cumulative impact of ongoing costs and some patients cannot afford any additional costs [16]. FT impacts a patients’ ongoing ability to afford medications, mortgages, family expenses, and may add to their overall distress.

## Conclusions

Australian HCPs believe that FT is a distressing issue for many cancer patients and an urgent, coordinated effort is required to limit its impact. As cancer care continues to evolve, FT supportive care efforts must also adapt and advance. Patients, survivors, families, and HCPs need to be empowered to navigate complex and life-altering financial decisions, insurance policies, and government agencies. Training our workforce to identify and address these needs is an urgent and critical challenge .

## Supplementary Information

Below is the link to the electronic supplementary material.

Supplementary file1 (DOCX 14 KB)

## Data Availability

Data is available on request from the first author in collaboration with the COSA Financial Toxicity Working Group.
