# Supplementary material for: Challenges and solutions to cancer-related financial toxicity according to Australian health professionals: qualitative results from a national survey
Source: Support Care Cancer. 2023 Jul 4;31(7):441. doi: 10.1007/s00520-023-07875-4 (PMC10319649; doi:10.1007/s00520-023-07875-4)
Supplement: Supplementary file 1 — Supplementary file1 (DOCX 14 KB) [file 520_2023_7875_MOESM1_ESM.docx]

Appendix A: Open-ended items presented to respondents completing the online survey

| 1. Would you like to provide any further information about financial concerns? 2. Would you like to comment further about who usually raises financial concerns? 3. Would you like to comment further about the time point of discussions? 4. Would you like to comment further about the ideal time to discuss concerns? 5. In your opinion, whose role is best-placed to help patients when financial concerns are a problem and why? 6. Would you like to comment further about potentially offending a patient? 7. Would you like to comment further about how comfortable you are raising financial concerns? 8. What other barriers to discussing financial concerns with your patients do you experience? 9. What other strategies do you use if the topic of financial difficulty is raised by your patient? 10. What other financial toxicity related information needs do you have? 11. What services are you aware of that provide financial support or advice to patients with cancer? (either within or external to your organisation)? 12. Please provide any other comments you have on financial toxicity that we have not addressed. These might include suggestions, needs, views about your clinical role, work you are currently undertaking, or practice/policy initiatives or ideas. |
| --- |
